# Mandatory vaccination and the ‘seat belt analogy’ argument: a critical analysis in the context of the Covid-19 pandemic

**DOI:** 10.1007/s11019-022-10068-1

**Published:** 2022-02-07

**Authors:** Iñigo de Miguel Beriain

**Affiliations:** 1 University of the Basque Country, Leioa, Bizkaia Spain; 2 Ikerbasque, Basque Foundation for Science, Bilbao, Spain

**Keywords:** Seat belt analogy, Coercive vaccination, Mandatory vaccination, Compulsory vaccination, Vaccination systems

## Abstract

The seat belt analogy argument is aimed at furthering the success of coercive vaccination efforts on the basis that the latter is similar to compulsory use of seat belts. However, this article demonstrated that this argument does not work so well in practice due to several reasons. The possibility of saving resources in health care does not usually apply in our societies, and the paternalist mentality that contributed to the implementation of seat belt–wearing obligation was predominant 30 years ago, but it does not apply at this moment. Furthermore, the risk/benefit analysis is totally different in both scenarios. In the case of seat belts, there is no way to discriminate between the users. In the case of vaccines, individuals present with unique circumstances that may differ substantially from those of another and might be foreseen a priori. This means that an analysis must be performed individually before vaccination is imposed. Finally, one must keep in mind that seat belts are often the only way in which we can protect third parties against a tragic hit by the occupant of another vehicle and are very efficient tools for this purpose. Vaccines, in contrast, do not always create sterilising immunity and are definitely not the only way by which we can avoid spreading a virus; immunity certificates, isolation, or even confinement may also serve as viable methods to achieve this purpose.

## Introduction

The debate about coercive vaccination in the context of the Covid-19 pandemic is gaining intensity in most Western countries. Currently, several factors may be combined to explain this phenomenon: on the one hand, authorities are just finishing the rollout of vaccines to all those sectors of the population that were eager to be immunised, while, on the other, the appearance of new variants with greater contagiousness, such as the delta variant, means that, in principle, it will be necessary to vaccinate a greater percentage of the total population to achieve herd immunity, which, in turn, is expected to intensify the debate on whether we should vaccinate children, including those younger than 12 years of age. Given such circumstances, it is not surprising that arguments both for and against coercive vaccination are proliferating (Giubilini et al. 2020; De Miguel Beriain 2021; Brusa and Barilan 2021).

One of the arguments that have been put forward in recent days (Singer 2021) in support of coercive vaccination against Covid-19 has been that of the ‘seat belt analogy (SBA)’, which Giubilini and Savulescu had already defended prior to the appearance of the severe acute respiratory syndrome coronavirus (Giubilini and Savulescu 2019). In a nutshell, this argument highlights that, 50 years ago, the gradual introduction of compulsory seat belt–wearing succeeded in changing social habits, preventing thousands of deaths that would otherwise have occurred. Since, in the opinion of its advocates, the situation posed by vaccines is analogous to that of seat belts, we should proceed to implement coercive vaccination policies on the basis of this historical experience with seat belts.

The purpose of the present article is, first, to show that not all arguments in favour of mandatory seat belts are as convincing as they seem at first glance. Second, it exposes that, although there are some strong reasons in favour of mandatory seat belt wear, they are not as sound when applied in the case of Covid vaccines, since the analogy between vaccines and seat belts is not as strong as its apologists believe it to be. To this purpose, it will be argued that, in the case of Covid vaccines, there are valid alternatives to achieve the same results that we wish to achieve through vaccines’ mandatory imposition. Similarly, the risk analysis of vaccination policies cannot imitate that of seat belt policies since risks associated with the different type of Covid vaccines vary considerably from one to another person. However, before analysing the analogy as such, a preliminary section is devoted to describing the seat belt analogy argument (SBAA) as such.

## The SBAA: a brief description

The Australian state of Victoria was the first jurisdiction in the world to make it compulsory to wear a seat belt in a car, in 1970. At that time, that legislation was attacked as a violation of individual freedoms. However, the excellent results obtained as a consequence made seat belt use coercive policies (SBUCPs) very popular in subsequent years. In most Western countries, seat belts laws have been enforced since the mid-1980s. According to some sources, these policies reduce the risk of death by 45 per cent and the risk of serious injury by 50 per cent (US. Dept. of Transporation 2009) or even more (Hoye 2016). These numbers encompass the harm that drivers, passengers and others may suffer from an accident; for instance, unbelted rear-seat occupants could slam into the front seat and push the driver into the airbag or steering wheel, a real scenario that enhances the risk by fivefold that the occupants of the front seats die in the accident (Ichikawa et al. 2002). Similarly, sometimes passengers are thrown out of the car due to the violence of the hit, causing damages to pedestrians or the occupants of another vehicle.

However, this does not necessarily mean that wearing a seat belt is always beneficial. It is undeniable that seat belts may cause direct injuries to those who wear them, such as skin abrasions of the neck or chest or perforation of the ileum and other internal organ damage requiring surgery to fix (the so-called ‘seat belt syndrome’) (Al-Ozaibi et al. 2016). In some cases, they might impede the escape of a person from a car on fire. However, the risk of these side effects (let us label them this way for the sake of the analogy) is definitely small. Thus, all a priori risk/benefit assessments clearly lean in favour of SBUCPs, no matter the individual physical conditions of those who wear the seat belt. This is particularly true if we also consider that wearing seat belts contributes to a good level of public health by saving health care resources that would otherwise have to be spent to treat injuries provoked by driving incidents. The final conclusion of all this evidence is clear: SBUCP have undoubtedly been successful in saving human lives and reducing the deployment of health care resources in this context (Hoye 2016).

What do SBUCPs have to do with coercive vaccination policies in the case of Covid? Authors such as Giubilini or Savulescu are somehow convinced that getting vaccinated or vaccinating one’s children against the virus is, in many respects, which are relevant for the ethics of policy-making, analogous to wearing a seat belt or to having one’s children wear seat belts. They wrote: ‘We can think of seat belts as a metaphor for vaccination: a vaccine protecting individuals against an infectious disease is like a seat belt protecting individuals in car accidents (…) Wearing a seat belt significantly reduces the risk that the car accident results in serious injury or death; in the same way, in the case of infectious diseases, vaccines significantly reduce the risk that exposure results in serious injury or even death’.

Thus, the position of those who support the consistence of the SBAA in the case of Covid is based on two main assumptions. First, they consider that the implementation of SBUCPs is undoubtedly justified since they help us to improve our situation by protecting people from the harm suffered in a car accident, avoiding injuries or deaths caused to third parties and/or saving some resources that would otherwise have to be spent treating people with injuries from car accidents. Second, they hold that the case of seat belts is similar to that of the Covid vaccines; in other words, what is applicable to the former must also be applicable to the latter. Their argumentation could be summarised in the following way: since compulsory use of seat belts is morally justified and those vaccines are similar to them, we should enact coercive vaccination policies (Giubilini and Savulescu 2019). However, is this really true? Are both assumptions as solid as the supporters of the SBAA state? It is time to explore them carefully.

## The legitimacy of SBUCPs and the issues related to similar policies in the health care context

Let us start by analysing the real moral consistency of SBUCPs and the reasons that are usually provided to justify them. First, the value of SBUCPs might be based on the savings they provide to public health systems, as mentioned above; however, this justification is not solid. On the one hand, because this argument is only applicable to those countries where public health systems exist, but, also, because citizens are not always required to follow certain behaviours or lifestyles in order to defend the interests of the society where they live. We never deny health care to patients whose adherence to treatment is very poor, despite the fact that this entails a great cost to the system, nor do we deny care to smokers, compulsive consumers of red meat, or those whose lifestyle habits are very unhealthy. It is true that currently there are discussions taking place to increase private health insurance for non-vaccinated people. For instance, Delta Air Lines have already announced that it will charge unvaccinated employees an additional $200 a month (Sullivan 2021). Furthermore, Singapore will stop covering Covid costs for those who decline to be vaccinated (Yoon 2021). However, these practices seem far from becoming a part of Western countries’ policies in public health care access.

Thus, forcing someone to vaccinate against Covid for these reasons would probably constitute unfair discrimination. One might, of course, argue that we are not introducing a good argument since nobody has talked about denying health care. We could instead introduce a tax that should only apply to people who refuse vaccination (similar to what happens with tobacco and alcohol, for instance). However, this would also constitute an exception to the general rules that we usually follow. We do increase the price of some products (not all toxic products—consider sugary beverages or red meat), but, in general, we do not overtax people because of their bad habits. In fact, if we implement such policies in the case of Covid-19, we would be introducing a novelty. This, of course, does not mean that we cannot proceed to do so. Indeed, we could even argue that circumstances change when we have to face a public health emergency. However, this would only separate us more from the case of SBUPCs, which are hardly connected to such a scenario (I will come back to the autonomy issue in the next sections of this paper).

However, one might reply that forcing someone to vaccinate against Covid on the basis of the need to preserve scarce resources makes sense, due to the special situation that we are living. In principle, this would make the SBUCPs more applicable to the case of vaccination. Indeed, the parallelism between the use of the seat belt and vaccines on the basis of preserving scarce resources does make sense when we are faced with a situation of a severe shortage of health resources, in which triage is imposed. Furthermore, it is perfectly reasonable to point out that both the seat belt and the vaccine not only protect those who use them, but also those who are most vulnerable, because they prevent someone else from unnecessarily occupying a scarce resource (such as a place in the ICU). The problem is that, in this case, the analogy could be extended to any risky activity that could be avoided: from paragliding to cycling downhill or skating. This makes the analogy somewhat meaningless.

On the other hand, we should always consider that if we are to accept that the need to save public resources in critical situations authorizes us to impose mandatory vaccination policies, this would open the door to a general restriction of individual autonomy in the health care arena. That is to say, if we force you to get vaccinated to optimize the use of public resources in times of scarcity, why couldn’t we use the same argument to force you to vaccinate against flu, or to undergo Covid curative treatment when it becomes available? Moreover, if health care resources are always scarce, why should this obligation be implemented only in times of pandemic? Why should we only introduce the obligation to vaccinate only in the case of Covid-19 and not in the case of influenza, for example? Or why shouldn’t we oblige patients to change their lifestyle as a whole? This would undoubtedly save resources that could benefit other patients.

As can be seen, the reasoning can give rise to serious problems in terms of respect for patient autonomy and even people’s choices concerning their lifestyles. Thus, it seems more reasonable to argue that seat belt policies are indeed useful to save public resources but, at the same time, are not extensible to the health care field because of the possible connotations. If we step outside this framework, we may fall back into a paternalism that we were right to banish in the first place. However, this leaves the analogy out of play, of course.

Second, SBUCPs are often based on a paternalistic attitude: we protect drivers and passengers even though they are not willing to protect themselves (most probably because we consider that they refrain due to an underperformance in terms of risk analysis). The problem, in this case, is that paternalism was an acceptable attitude in all areas of life until the 1980s, when seat belts were made compulsory. Indeed, it was only in the 80 s that the emergence of the shared decision-making model acknowledged the legitimate roles for both patients and physicians in the autonomy model (Siegler and Entralgo 2011). In the following forty years, circumstances have changed (Flanigan 2017). Though we continue to accept these practices in the case of driving (think not only of seat belts but also of motorcycle helmets), the scenario has changed substantially in the realm of health care. In fact, today, the paradigm ruling physician–patient relations is that of informed consent: treatments are no longer imposed on the patient on the grounds of his or her best interest as perceived by another; instead, it is now the patient who makes the decision to be treated or not, so much so that they can perfectly well refuse, for example, an amputation, even if such decision causes their death. This evolution should not be forgotten when drawing analogies between seat belts and vaccines, at the risk of falling into unacceptable paradoxes. Let us think, for example, that one of these days (fingers crossed) an effective treatment for coronavirus disease 2019 is discovered. Curiously, an infected patient could refuse to be treated, and nobody could impose vaccination on this patient on the basis of paternalism. One might reply to this argument by saying that, even in this case, people could be coercively vaccinated to avoid risks to third parties, but this is just a way of recognising that the justification of such conduct would not be related to paternalism but rather to the need to protect someone else’s health—a different topic that will be analysed in the following sections. On the other hand, one might consider that a return to paternalism could be acceptable in this case. However, this would not be a good idea. If we accept the permissibility of seat belt mandates, as Joel Feinberg once said, “the trick is stopping short once we undertake this path, unless we wish to ban whiskey, cigarettes, and fried foods”. (Feinberg 1971).

Of course, Giubilini and Savulescu would probably reply to this argument by saying that, at least, paternalism should be respected if we are thinking about children’s interests (Giubilini and Savulescu 2019). And, indeed, it is true that children constitute a clear exception against individual autonomy prevalence in health care: if children are at risk, they receive medical treatment, even if their parents do not provide their consent. The case of Jehovah’s Witnesses is enlightening in this regard: while we allow adult believers to refuse a transfusion for reasons of conscience, we do not allow this refusal to extend to minors under their custody. The key issue here is the risk/benefit analysis that we need to perform before an intervention. However, does risk analysis work the same in the case of the seat belts and that of the vaccines?

## Exploring the issues related to risk analysis: the differences between seat belts and vaccines

SBUCPs can easily be defended by performing a risk/benefit analysis of wearing the seat belt. Supporters of coercive vaccination policies usually consider that this applies exactly the same way in the case of vaccines: the risk associated with vaccination is clearly lower than the corresponding benefit (i.e., avoiding death or severe damage from a disease); thus, vaccination requirements should be imposed following the same rationale. However, there are some gross mistakes in this argument.

It is, indeed, undeniable that, in both cases—vaccination and seat belts—the risks are usually outweighed by the benefits (Pierik 2018). The main difference, however, is that, in the case of seat belts, this is not only true at the level of big data—a whole population—but also at the level of the concrete individual. It is true that forcing a particular population to wear seat belts will lead to better outcomes for that population as a whole, but it will also provide better protection for each of its individual members. In the case of vaccines, on the contrary, it is certain that their imposition will improve the situation in terms of the population (in that fewer people will die or have serious sequelae), but this is not necessarily the truth for each individual (for some, it is possible that not being vaccinated would be preferable). This is due to a very simple reason: unlike seat belts, vaccines are biological tools that interact differently with each person’s body; therefore, the variability of results grows exponentially. Most people benefit the same of seat belts (people who have obesity might benefit a little bit less than the rest (Reed et al. 2012)). The side effects of vaccination, in fact, vary greatly depending upon the circumstances of a concrete individual. Moreover, the scenario to be avoided is also much more varied. The severity of a car accident does not depend too much on the individual characteristics of the driver or passengers, but the severity of an illness does. Another essential difference is that both the possible effects of vaccines and the course of a disease in a particular patient can at least be intuited a priori, something that does not happen with traffic incidents. If we think of coronavirus disease 2019, for example, a diabetic, aged, or severely obese person is not at the same level of risk as someone who does not have any of these characteristics (Fagard et al. 2021).

Similarly, with some of the vaccines, at least, it does not appear that the side effects are the same regardless of whether the patient is a 70-year-old man or a young woman in her 30 s (Ledford 2021) nor, of course, are the risk levels the same between a patient who is allergic to some of the excipients in the vaccine and one who is not. Indeed, according to the CDC, anyone who has a known severe allergy (e.g., anaphylaxis) to any of the vaccine ingredients should not receive that vaccine (CDC 2021). Last, but not least, the different types of Covid vaccines have different risk/benefit profiles in terms of both side effects and efficiency. For instance, women aged 18–49 years might face an increased risk for TTS thrombocytopenia syndrome if they take a Janssen COVID-19 booster (CDC 2021). Countries such as Denmark ceased giving *the* Oxford-*AstraZeneca* Covid vaccine amid concerns about rare cases of blood clots that might override the benefits provided by the vaccine, at least in the case of young women. In addition, both Denmark and Sweden have suspended the use of the Moderna vaccine for people under the ages of 18 and 30, respectively, on the basis of a potential small increase in the risk of myocarditis and pericarditis among young adults (Paterlini 2021).

All this means that the analogy between seat belts and vaccines is not valid if we concentrate on the validity of the risk/benefit analysis, and, crucially, this becomes much more complicated in the case of the latter. As a general rule, the assessment must, as far as possible, be individualised for each patient since individual conditions are key, yet this is not at all the case with seat belts. Hence, the argument that we should impose vaccination for the same reason of risk reduction for which we force people to wear seat belts is not nearly as valid as it might seem at first glance. Instead, we should consider the circumstances of each concrete person or, at least, group of persons, since Covid does not usually work the same way if we compare elderly and younger people, or people with diabetes and healthy people. Moreover, the different types of Covid vaccines do not offer the same risk/benefit relation for each population group or even concrete individuals. This constitutes a dramatic difference in terms of the implementation of the policies.

## Assessing the benefits to third parties: does the analogy work when we consider the risk of harm to others?

Let us now concentrate on the a priori most convincing part of the analogy: both seat belts and vaccines should be compulsory because they help to protect third parties’ lives. At first, this sounds like the definitive argument; however, in practice, it does not fit so perfectly. First, what the analogy forgets is that there are major differences between the benefits obtained and the way they are achieved in the case of vaccines and in the case of seat belts, respectively. Considering seat belts, the benefit to third parties does not depend upon what others do. In other words, if one wears a seat belt, it should prevent them from being thrown against the front seat occupants, regardless of what the other occupants in the vehicle do. In the case of vaccines, however, effective protection of those third parties will only be achieved if a lot of people get vaccinated—that is, if the society can collectively at least get close to achieving herd immunity (De Miguel Beriainand Rueda 2020, 2021; Brown et al. 2020). If this is not the case, it is likely that a single individual’s attitude, by itself, will not serve to prevent others from becoming infected. Thus, this clearly creates a substantial difference: vaccines require collective actions that belts do not demand. Of course, this reasoning does not mean that the argument for coercive vaccination is weakened (indeed, if herd immunity is needed and individual action is not so useful, the argument is reinforced), but it does debunk the inconsistency in the analogy.

Second, we must also bear in mind that seat belts, in general, are the only reasonable option we have to protect third parties in a car. We can protect a driver or the occupant of the front passenger seat with dump air bags. However, there is little we can do to protect them if they are hit from behind by a passenger sitting in the rear seats, other than to oblige the latter to fasten their seat belts. In the case of virus transmission to third parties, instead, there are multiple ways to achieve this goal, ranging from the imposition of immunity certificates, already widely advocated by academia and governments (Giubilini and Savulescu 2019), to the confinement of people (as New Zealand, for instance, is still promoting (Hollingsworth and Thornton 2021)). On the other hand, it is quite clear that the strength of the argument depends upon the capability of the vaccines to substantially impede the dissemination of the virus. In other words: if vaccines do not provide substantial sterilising immunity but instead only prevent more serious presentations of the disease, then mandatory vaccination will not prevent harm to others. These differences involve severe consequences in terms of moral obligations: if someone makes compromises to avoid spreading the virus by, for instance, monitoring his or her health on a daily basis, this could be as helpful as getting vaccinated. This is a circumstance that can hardly apply in the case of seat belts: one cannot offer an alternative to this tool (Mina et al. 2021).

Therefore, one must conclude that there are significant differences in the way mandatory vaccine and seat belt policies can be justified on the basis of avoiding harm to others. If this might be true (even not so important) in the case of seat belts, it is not necessarily true when vaccines are involved. Indeed, a number of circumstances must function together to ensure that vaccination is a perfectly adequate and necessary tool to prevent the spread of a virus. Thus, the analogy would only apply in some concrete circumstances as follows: the vaccines confer sterilising immunity, herd immunity is at reach, no other tools devoted to preventing virus spread are applicable, etc. To sum up, many circumstances must concur in order to consider that the posed analogy really works well in practice.

## Data Availability

Not applicable.
